# Fiber optical shape sensing of flexible instruments for endovascular navigation

**DOI:** 10.1007/s11548-019-02059-0

**Published:** 2019-09-06

**Authors:** Sonja Jäckle, Tim Eixmann, Hinnerk Schulz-Hildebrandt, Gereon Hüttmann, Torben Pätz

**Affiliations:** 1 Fraunhofer MEVIS, Institute for Digital Medicine, Lübeck, Maria-Goeppert-Straße 3, 23562 Lübeck, Germany; 2 Medical Laser Center Lübeck GmbH, Peter-Monnik-Weg 4, 23562 Lübeck, Germany; 3 Institute of Biomedical Optics, Universität zu Lübeck, Peter-Monnik-Weg 4, 23562 Lübeck, Germany; 4 German Center for Lung Research, DZL, Airways Research Center North, 22927 Großhansdorf, Germany; 5 Fraunhofer MEVIS, Institute for Digital Medicine, Bremen, Am Fallturm 1, 28359 Bremen, Germany

**Keywords:** Fiber Bragg grating (FBG), Shape sensing, Flexible instruments, Endovascular navigation

## Abstract

**Purpose:**

Endovascular aortic repair procedures are currently conducted with 2D fluoroscopy imaging. Tracking systems based on fiber Bragg gratings are an emerging technology for the navigation of minimally invasive instruments which can reduce the X-ray exposure and the used contrast agent. Shape sensing of flexible structures is challenging and includes many calculations steps which are prone to different errors. To reduce this errors, we present an optimized shape sensing model.

**Methods:**

We analyzed for every step of the shape sensing process, which errors can occur, how the error affects the shape and how it can be compensated or minimized. Experiments were done with one multicore fiber system with 38 cm sensing length, and the effects of different methods and parameters were analyzed. Furthermore, we compared 3D shape reconstructions with the segmented shape of the corresponding CT scans of the fiber to evaluate the accuracy of our optimized shape sensing model. Finally, we tested our model in a realistic endovascular scenario by using a 3D printed vessel system created from patient data.

**Results:**

Depending on the complexity of the shape, we reached an average error of 0.35–1.15 mm and maximal error of 0.75–7.53 mm over the whole 38 cm sensing length. In the endovascular scenario, we obtained an average and maximal error of 1.13 mm and 2.11 mm, respectively.

**Conclusion:**

The accuracies of the 3D shape sensing model are promising, and we plan to combine the shape sensing based on fiber Bragg gratings with the position and orientation of an electromagnetic tracking to obtain the located catheter shape.

**Electronic supplementary material:**

The online version of this article (10.1007/s11548-019-02059-0) contains supplementary material, which is available to authorized users.

## Introduction

Cardiovascular diseases are the main cause of death in western industrial nations [9]. Some of these diseases like abdominal aortic aneurysms can be treated with an endovascular aortic repair (EVAR) procedure, in which a stent graft is placed in the aneurysm region under 2D fluoroscopy. To reduce the X-ray exposure and to supersede the angiography, a three-dimensional navigation is needed.

Fiber Bragg grating (FBG)-based systems are used for shape sensing, which enables three-dimensional navigation. FBGs are interference filters inscribed into the core of an optical fiber, which reflect a specific wavelength. Combining multiple FBGs at the same longitudinal position allows to calculate curvature and direction angle. The most common configurations are three fibers arranged triangular around the structure to be measured [4, 13]. This introduces significant errors due to possible changes in the core geometry [4], which can be overcome with multicore fibers, where several cores are integrated into one fiber [10]. In addition, other FBG systems with other geometries have been introduced, for instance helically wrapped [17].

Most research groups use FBG systems for shape and force sensing of medical needles [11], which have a simple bending profile allowing only shapes with low bending and no torsion. For example, Park [12] applied FBGs for shape sensing of biopsy needles and Roesthuis used it to reconstruct the shape of a nitinol needle [13]. A few works using optical fibers for flexible instruments have been reported in the literature: Shi [15] used FBGs for catheter shape sensing. Also Khan [7] used four multicore fibers to reconstruct the first 118 mm of a catheter. However, to our knowledge, there are currently no studies on the accuracy of fiber optical shape sensing for very long and flexible medical instruments.

In general, shape reconstruction of flexible structures is more challenging, because higher deflections and torsion can occur. Thus, the error analysis of the shape reconstruction from measured wavelengths to the reconstructed shape becomes more important. Also, the shape accuracy has to be very accurate, since the error accumulates along the fiber.

In each shape sensing step, the errors were analyzed to determine how strongly the preserved form is influenced and how it is best reconstructed. Using this, we optimized our shape sensing model by compensating or minimizing the various error sources. Then, we evaluated our optimized model with 3D shape measurements. Finally, we tested it in a realistic endovascular scenario by inserting our fiber in a 3D printed vessel.

**Fig. 1:**
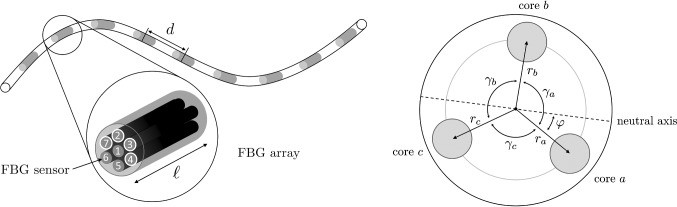
Left: a FBG system with center-to-center distance *d* and sensor length $\ell$. The different cores are represented by numbers: here the cores of configuration (2347) are highlighted. Right: the cross section of a triplet configuration

## Material and methods

We consider a multicore fiber with *n* FBG arrays along a flexible instrument, as shown in Fig. 1. Each array contains seven FBGs, one center core and six outer cores. All FBGs have fixed length $\ell$, and the arrays are uniformly distributed with center-to-center distance *d*.

### Shape sensing model

We analyzed every shape sensing step and optimized it by minimizing the errors. The result is our optimized shape sensing model:

1. Wavelength shift calculation.
2. Strain computation for every core.
3. Strain interpolation for every core.
4. Curvature and angle calculation.
5. Curvature and angle correction.
6. Shape reconstruction with circle segments.

Every step is described in more detail in the following sections.

#### Wavelength shift calculation

FBGs are interference filters inscribed in short segments of an optical fiber core, which reflect a specific wavelength of the incoming light [8]. The Bragg wavelength of a FBG is defined as

$$\begin{aligned} \lambda _{\mathrm{{B}}} = 2n_{\mathrm{{e}}} \Lambda , \end{aligned}$$

where $n_\mathrm{e}$ is the effective refractive index of the grating and $\Lambda$ the grating period. Mechanical strain or temperature change influence the reflected wavelength. This results in a wavelength shift

$$\begin{aligned} \Delta \lambda = \lambda - \lambda _\mathrm{B} \end{aligned}$$

of the measured wavelength $\lambda$ in comparison with the reference wavelength $\lambda _\mathrm{B}$ of the FBG. If the reference wavelengths of the FBGs are unknown, they have to be determined by a separate measurement where no strain is applied to the fiber system.

#### Strain computation

The measured wavelength shift $\Delta \lambda _\mathrm{B}$, which can be caused by applying strain $\varepsilon$ or by changing temperature $\Delta T$ in the Bragg gratings, is given by

$$\begin{aligned} \Delta \lambda = \lambda _\mathrm{B} \big ((1-p_\mathrm{e})\varepsilon +(\alpha _\Lambda + \alpha _n)\Delta T\big ), \end{aligned}$$

where $p_\mathrm{e}$ is the photo-elastic coefficient and $\alpha _\Lambda$ and $\alpha _n$ are the thermal expansion coefficient and the thermo-optic coefficient [5]. By assuming a constant temperature $\Delta T = 0$ the applied strain of the FBG can be calculated:

$$\begin{aligned} \Delta \lambda _\mathrm{b} = \lambda _\mathrm{b} (1-p_\mathrm{e})\varepsilon . \end{aligned}$$

The photo-elastic coefficient $p_\mathrm{e}$ is directly related to the gauge factor $GF = 1 - p_\mathrm{e}$. Photoelasticity is defined as the change in reflected wavelength depending on the strain applied in axial direction. For FBG systems, the photo-elastic coefficient $p_\mathrm{e} \approx 0.22$ can be found in the literature [16] and experiments for determining the parameter of any FBG system are described [2].

#### Interpolation

When the curvatures and angles are calculated for every FBG array, the intermediate values can be determined by interpolation. Henken [4] compared common interpolation methods for shape sensing and concluded that cubic spline interpolation is the best solution, which is currently the state-of-the-art interpolation. Interpolating the curvature is straight forward since it is continuous for every shape, whereas the angle interpolation is challenging for flexible structures, which may have discontinuous angle. Thus, we suggest to interpolate the strain, since it is continuous.

Furthermore, it is assumed that the measurements of one FBG array are the values for one specific position, usually the array center. Thus, we use the averaged cubic interpolation, as introduced in [6]: this yields a realistic interpolation based on the spatial properties of a FBG by modeling the measured value as an average over the sensor range.

#### Curvature and angle computation

The calculation of the curvature and direction angle depends on the fiber system. The most common one is a triplet configuration [4, 13]: here, the FBG system has three fiber cores with specific angles (typically $120 ^\circ$) in between, as illustrated in Fig. 1.

For this configuration, the relationship between strain, curvature and directional angles is described by the following equations:

1 $$\begin{aligned} \begin{aligned} \varepsilon _\mathrm{a}&= - \kappa r_\mathrm{a} \sin (\varphi ) + \varepsilon _0 \\ \varepsilon _\mathrm{b}&= - \kappa r_\mathrm{b} \sin (\varphi + \gamma _\mathrm{a}) + \varepsilon _0 \\ \varepsilon _\mathrm{c}&= - \kappa r_\mathrm{c} \sin (\varphi + \gamma _\mathrm{a} + \gamma _\mathrm{b}) + \varepsilon _0, \end{aligned} \end{aligned}$$

where $\varepsilon _x$ is the strain, $r_x$ the radius and $\gamma _x$ the angle of fiber *x*. By solving the equation system, we obtain the strain bias $\varepsilon _0$, the curvature $\kappa$ and the direction angle $\varphi$. The equation system can also be extended for four or more fibers.

The equations show that the curvature is influenced by the radii $r_x$ similarly as by the photo-elastic coefficient. In addition, the strain is also biased by a temperature change, additional axial strain and pressure. Due to the short distance ($< 100~ \upmu \mathrm{m}$) of the FBGs in one array, it can be assumed that for every grating in one array (see Fig. 1), this bias is equal and therefore compensated by the strain bias $\varepsilon _0$.

#### Curvature and angle correction

The determined curvatures and angles are influenced by various variables; therefore, we suggest the following corrections: the curvatures are scaled by the photo-elastic coefficient $p_\mathrm{e}$ and the center-to-core distances $r_x$. Since both parameters can be biased, we determine a correction parameter *c* to get the right curvatures

2 $$\begin{aligned} \kappa _{\text {real}} = c \cdot \kappa . \end{aligned}$$

This factor must be determined individually for each fiber. Also, the fiber can be twisted during production or storage, but these twists are not contained in $\varepsilon _0$. Thus, we obtain a measured angle

3 $$\begin{aligned} \varphi = \varphi _\text {real} + \varphi _\text {twist}, \end{aligned}$$

which does not equal the real angle $\varphi _\text {real}$ because it is distorted by the twist angle $\varphi _\text {twist}$. The twist angle $\varphi _\text {twist}$ cannot be determined for fibers of this geometry without a measurement, where $\kappa \ne 0$ [see also Eq. (1)]. Helically wrapped fibers include torsion in their model, and the twist can be calculated. For short and stiff instrument, this error is negligibly, whereas for flexible instruments the twist angles must be determined.

#### Shape reconstruction

In the last years, three shape reconstruction algorithms have been proposed: Moore [10] presented a method based on the fundamental theorem of curves, which states that the shape of any regular three-dimensional curve with nonzero curvature can be determined by its curvature and torsion [1].In mathematical contexts, the torsion of curves corresponds to the change of the direction angle. The shape is obtained by solving the Frenet–Serret equations:

$$\begin{aligned} \frac{\mathrm{d}T}{\mathrm{d}t} = \kappa N, \; \frac{\mathrm{d}N}{\mathrm{d}t} = - \kappa N + \tau B, \; \frac{\mathrm{d}B}{\mathrm{d}t} = -\tau N, \end{aligned}$$

where $\kappa$ is the curvature, $\tau$ the torsion, *T* the tangent vector, *N* the normal vector and *B* the binormal vector of the curve at length position *t*. The integration of the determined tangent vectors yields the shape of the curve. This method fails at points with $\kappa = 0$ since the torsion is undefined there. Thus, this algorithm is not suitable for shape sensing of flexible structures.

Cui [3] suggested a method based on parallel transport to overcome this problem. The equations to be solved are:

$$\begin{aligned} \frac{\mathrm{d}T}{\mathrm{d}t} = \kappa _1 N_1 + \kappa _2 N_2, \; \frac{\mathrm{d}N_1}{\mathrm{d}t} = - \kappa _1 T, \; \frac{\mathrm{d}N_2}{\mathrm{d}t} = -\kappa _2 T. \end{aligned}$$

where $\kappa _1$ and $\kappa _2$ are the curvature components corresponding to the normal vectors $N_1$ and $N_2$, which are orthogonal to the tangent vector *T*. The shape reconstruction is conducted in the same way as with Frenet–Serret.

Roesthuis [13] proposed another method based on circle segments: the shape is reconstructed by approximating it with elements of constant curvature. For every element a circle segment of curvature $\kappa$ and length *l* is created and is rotated by the direction angle $\varphi$. By repeating this procedure for every given set $(\kappa , \varphi )$ we obtain the whole shape.

### Experimental methods

For all experiments described below we had one multicore fiber system (FBGS Technologies GmbH) available with 7 cores, one center core and six outer cores with an angle of $60^{\circ }$ in between, as shown in Fig. 1. It has 38 FBG arrays each with 5 mm length and 10 mm center-to-center distance, which are chains of draw tower gratings (DTG$\circledR$).

In the next sections, we used the following parameters and algorithms if they are not analyzed or specified otherwise: we fixed our covered fiber to a precise ruler and used the measured wavelength as reference wavelengths, we used a photo-elastic coefficient $p_\mathrm{e} = 0.22$, made averaged cubic strain interpolation, used four outer cores and reconstructed the shape with circle segments.

For matching reconstructed and ground truth shape, we used the iterative closest point algorithm [14]. For evaluation, we calculated the average and maximum error defined as

$$\begin{aligned} e_{\text {avg}}:= & {} \frac{1}{n}\sum _{i=0}^{n} \Vert x_i - x^{\text {gt}}_i \Vert _2 \text { and }\\ e_{\text {max}}:= & {} \max ( \Vert x_0 - x_0^{\text {gt}} \Vert _2, \ldots , \Vert x_n - x_n^{\text {gt}} \Vert _2), \end{aligned}$$

where $x_0, \ldots , x_n$ are the reconstructed points and $x_0^{gt}, \ldots , x_n^{gt}$ are the ground truth points located every $10 \, \text {mm}$ along the shape.

#### Wavelength shift computation

For our multicore fiber, we had no reference Bragg wavelengths given. Thus, we had to determine these wavelengths with a measurement without any strain. Therefore, we analyzed the effect of the Bragg wavelength estimation: at different times we fixed the fiber in a straight line, measured the wavelengths, used it as reference Bragg wavelengths and reconstructed various types of shapes.

#### Strain calculation

The photo-elastic coefficient influences the shape by curvature scaling. To analyze this effect, we bent our fiber to varying degrees and reconstructed the shape with different $p_\mathrm{e}$ values.

**Table 1:** Results of the Bragg wavelength study: measured errors $e_{\text {avg}}$ and $e_{\text {max}}$ in mm for different shapes using various Bragg wavelengths

Shape	Error	First reference	Second reference	Third reference
Straight line	$e_{\text {avg}}$	0.36	0.16	2.28
	$e_{\text {max}}$	1.00	0.30	5.49
Bended curve	$e_{\text {avg}}$	1.70	1.56	1.59
	$e_{\text {max}}$	4.92	4.53	4.82
S-curve	$e_{\text {avg}}$	1.80	1.76	1.38
	$e_{\text {max}}$	4.58	4.62	3.06

#### Interpolation

We formed our fiber to a snakelike shape, which has a few singularity points, interpolated the measured strains as proposed in “Interpolation” section and compared the resulting curvature and angles with the ones of common interpolation methods.

#### Curvature and angle computation

Since we have a multicore fiber with six outer cores and one center core and an interrogator, where we can connect four cores, we do not have to use a triplet configuration with $120^{\circ }$ in between. Thus, we analyzed the effect of different core configurations on the resulting curvatures and angles.

#### Curvature and angle correction

To determine the twist angle $\varphi _{\text {twist}}$, we bent our fiber to a 2D-shape, where every position has the same angle and used the determined angles as twist angles, as described in Eq. (3). To get the curvature scale factor *c* of our fiber, we made bow shapes with different radii, determined the best value assuming a photo-elastic coefficient $p_\mathrm{e} = 0.22$ and used it for curvature correction, as described in Eq. (2).

#### Shape reconstruction

The shape reconstruction quality depends completely on the input: when the measured values are correct, the proposed algorithms can reconstructed the correct shape. Therefore, we analyzed the following two aspects:

First, we looked at the convergence, i. e. how fine the segments in each step must be for accurate shapes. Second, we analyzed the noise handling of the three algorithms, i. e. how the resulting shape change with increasing Gaussian noise. In both cases, we simulated an arc shape with torsion, calculated the average curvature and median angle for every segment and reconstructed the shape.

#### 3D shape reconstruction accuracy

To evaluate our model, we recorded 3D measurements: we covered our fiber (diameter: $200\,\upmu \mathrm{m}$) with a metallic capillary tube (inner diameter: $300\,\upmu \mathrm{m}$, total diameter: $400\,\upmu \mathrm{m}$), fixed it in a specific shape, computed the shape and compared it with the segmented ground truth from the CT image. For the endovascular experiment, we inserted our fiber into a 3D printed vessel, which was created from a CT patient scan.

## Results and discussion

### Wavelength shift calculation

Table 1 shows the shape accuracies which were reconstructed with different reference wavelengths. The used wavelengths were determined experimentally by placing the fiber as straight as possible. For all three shapes, the obtained errors differ by several millimeters. This indicates that the reference wavelengths measurement has to be very accurate to obtain reconstructed shapes with a high accuracy. Thus, we recommend to determine the reference wavelengths by fixing it to a precise ruler, because here no kind of strain is applied.

### Strain computation

**Fig. 2:**
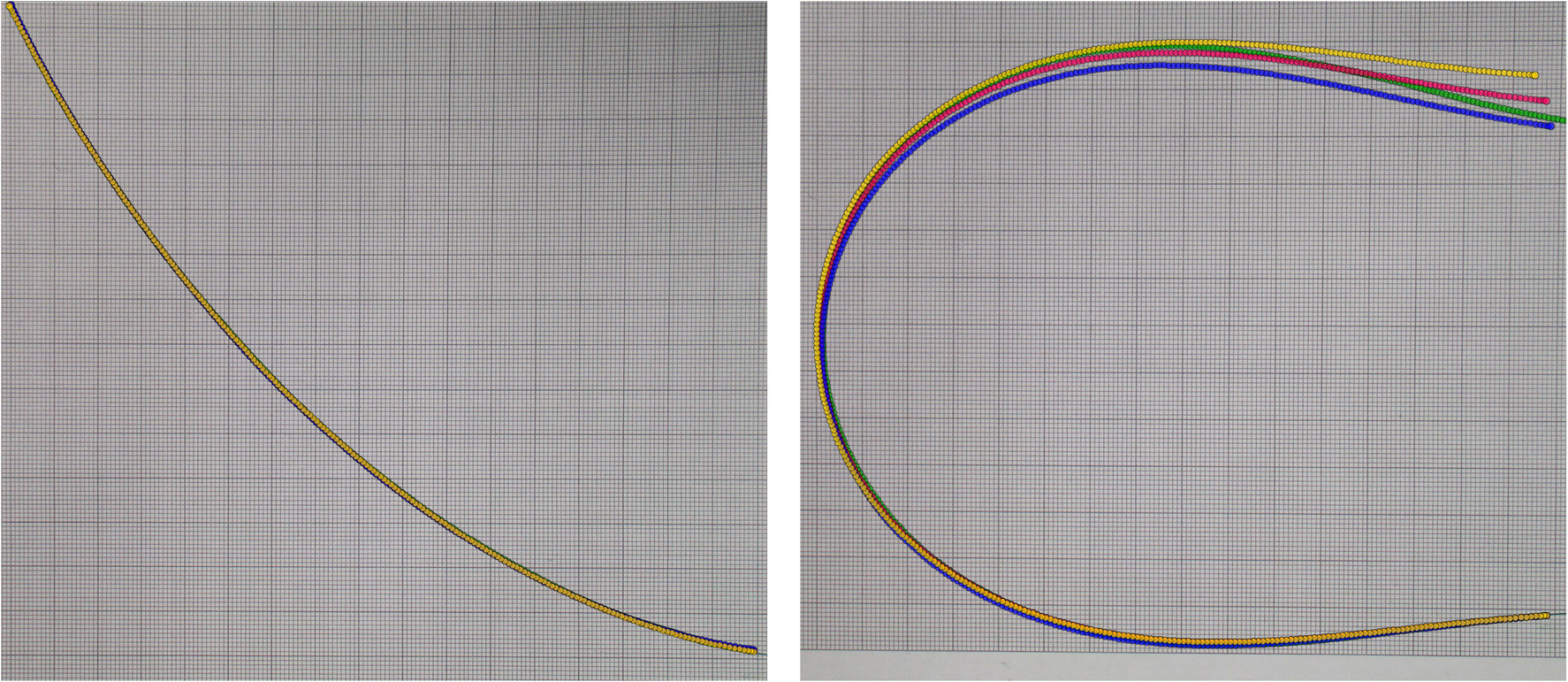
Effect of the photo-elastic coefficient for different bendings [ground truth (green), reconstruction with $p_\mathrm{e}=0.21$ (yellow), $p_\mathrm{e}=0.22$ (red) and $p_\mathrm{e} = 0.23$ (blue)] : the images show the fiber with low (first image) and high (second image) bending and the inserted projections of the reconstructed shape

The effect of the photo-elastic coefficient is shown in Fig. 2 for shapes with different bending strengths. In the left image, the reconstructed shape does not change notably, whereas in the right image the reconstructions differ significantly. Thus, the photo-elastic coefficient has high effects for high curvatures, while it has a minor effect on slightly curved structures. Therefore, it is important to determine the right photo-elastic coefficient or to compensate this error by determining the scale factor in another experiment to get accurate reconstructions.

### Interpolation

**Fig. 3:**
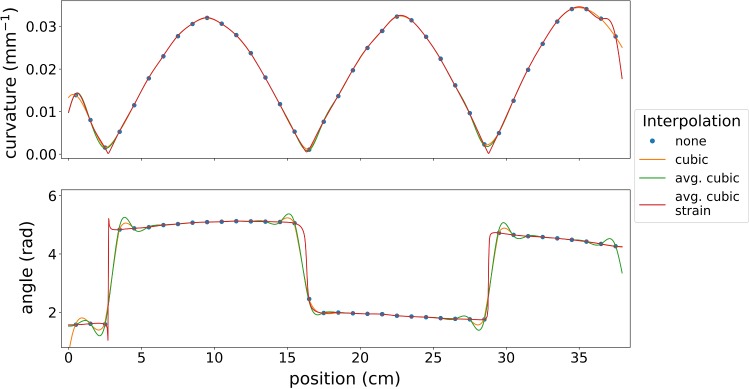
Results of interpolation study: the images show the resulting curvatures and angles of different interpolations methods along the fiber

For interpolation evaluation, we used a snakelike shape, which has discontinuous direction angles. The resulting curvatures and angles of our average cubic strain interpolation and the state-of-the-art methods are shown in Fig. 3. Interpolating the strain instead of curvature and angle leads to more accurate interpolation: at discontinuity points the curvature is closer to zero and the angles are more accurate, whereas interpolating the angle results in overshoots before and after the discontinuity, which can result in worse reconstructions. Thus, we recommend to interpolate the strain with our interpolation method, since it leads to most realistic curvatures and angles and in the end to more accurate shapes. The interpolation influence on the shape accuracy depends on the center-to-center distance *d*: a higher distance of the FBG arrays leads to more values that must be interpolated. For the distance of our fiber, the effects were low, see also [6].

### Curvature and angle computation

We calculated the curvature and direction angles using first a configuration with three cores (234), a configuration with three cores (347) and a configuration with four cores (2347). The numbers correspond to the cores as shown in Fig. 1, where the cores of configuration (2347) are highlighted in white. We observed that configuration (347) leads not to sufficient results due to the linear dependency of two cores. For proper 3D reconstruction, at least 3 linear independent cores are needed as observed with configuration (234) and (2347). The performance of FBGs in different cores in the multicore fiber varies quite significantly due to the manufacturing process. One side of the fiber will perform better, and this is why an asymmetrical configuration is preferable. Nonetheless due to low signals for shapes with high local bending radii, it is recommended to use more cores to ensure functionality for all possible shapes. Another possibility is to calculate multiple local strains for differing configurations and thus enable local averaging leading to higher accuracies.

### Curvature and angle correction

**Fig. 4:**
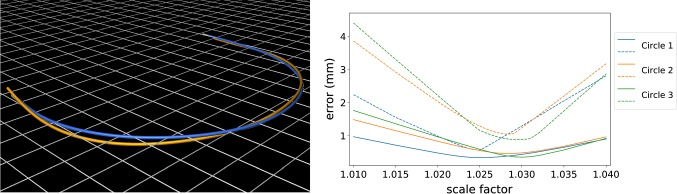
Left: results of the twist angle study: the reconstructions with twist correction (blue) and without (orange) are shown. Ground truth from the CT scan is displayed in white with the plane shown as grid; Right: results of the curvature scale study: the average error (straight line) and maximum error (dashed line) are plotted for three circles

First, we bent the fiber to a bow, where $\kappa \ne 0$, and used the determined angles for the angle correction, as described in Eq. (3). The result of this experiment is displayed in the left image of Fig. 4: the reconstruction without correction is twisted, whereas the corrected shape lies in the plane of the ground truth and is much more accurate. Afterward, we made several circular shapes with various radii to determine the curvature scale factor of our fiber. The results are shown in the right image of Fig. 4: we found that a scale factor of $\approx 1.026$ achieves the best results and used it for curvature correction of our fiber, as described in Eq. (2). With both corrections, we obtain now a high reconstruction accuracy, as shown in Table 2. However, these corrections allow a compensation of errors that do not change over time; other errors like dynamic twist are not corrected.

**Table 2:** Results of the 3D experiment: measured errors $e_{\text {avg}}$ and $e_{\text {max}}$ in mm for different 3D shape measurements using our shape sensing model with and without the curvature and angle correction

Shapes${\setminus }$errors (mm)	With corrections		Without corrections	
	$e_{\text {avg}}$	$e_{\text {max}}$	$e_{\text {avg}}$	$e_{\text {max}}$
Circle 1	0.35	0.75	3.53	12.57
Circle 2	0.50	1.15	4.91	18.13
Circle 3	0.50	1.02	5.70	23.66
S-curve 1	0.70	1.29	5.77	17.83
S-curve 2	0.57	1.98	1.78	4.27
S-curve 3	1.15	7.53	2.33	6.57
Helix	1.00	4.72	6.04	25.72
Inside the vessel	1.13	2.11	2.59	7.00

### Shape reconstruction

**Fig. 5:**
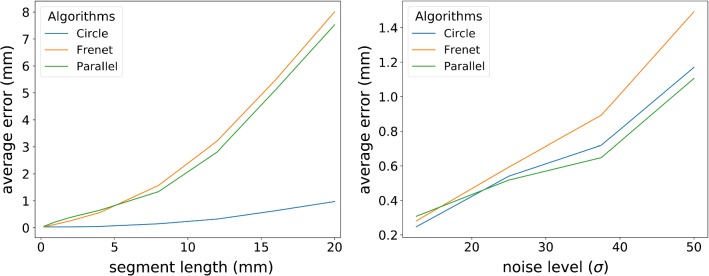
Shape reconstruction study results: the images show the average error as a function of segment length and of noise

The results of the convergence study are summarized in the left image of Fig. 5: the method based on circle segments has a faster convergence than Frenet–Serret and parallel transport. A reason for that might be, that for Frenet–Serret and parallel transport the differential equations have to be solved to get the tangent vectors, whereas for circle segments the shape is directly reconstructed. In the right image of Fig. 5, the noise study results are shown: the methods based on parallel transport and circle segments have significantly better noise handling than Frenet–Serret, which indicates that both methods are more stable and less prone to faults than Frenet–Serret. Considering the results of both experiments, we used the circle segment approach as shape reconstruction method. Parallel transport is also suitable, but the segment length has to be chosen fine enough ($< 5~\mathrm{mm}$) to get accurate reconstructions, whereas Frenet–Serret is not suitable, since it cannot reconstruct flexible structures as s-curves and has the worst noise handling.

**Fig. 6:**
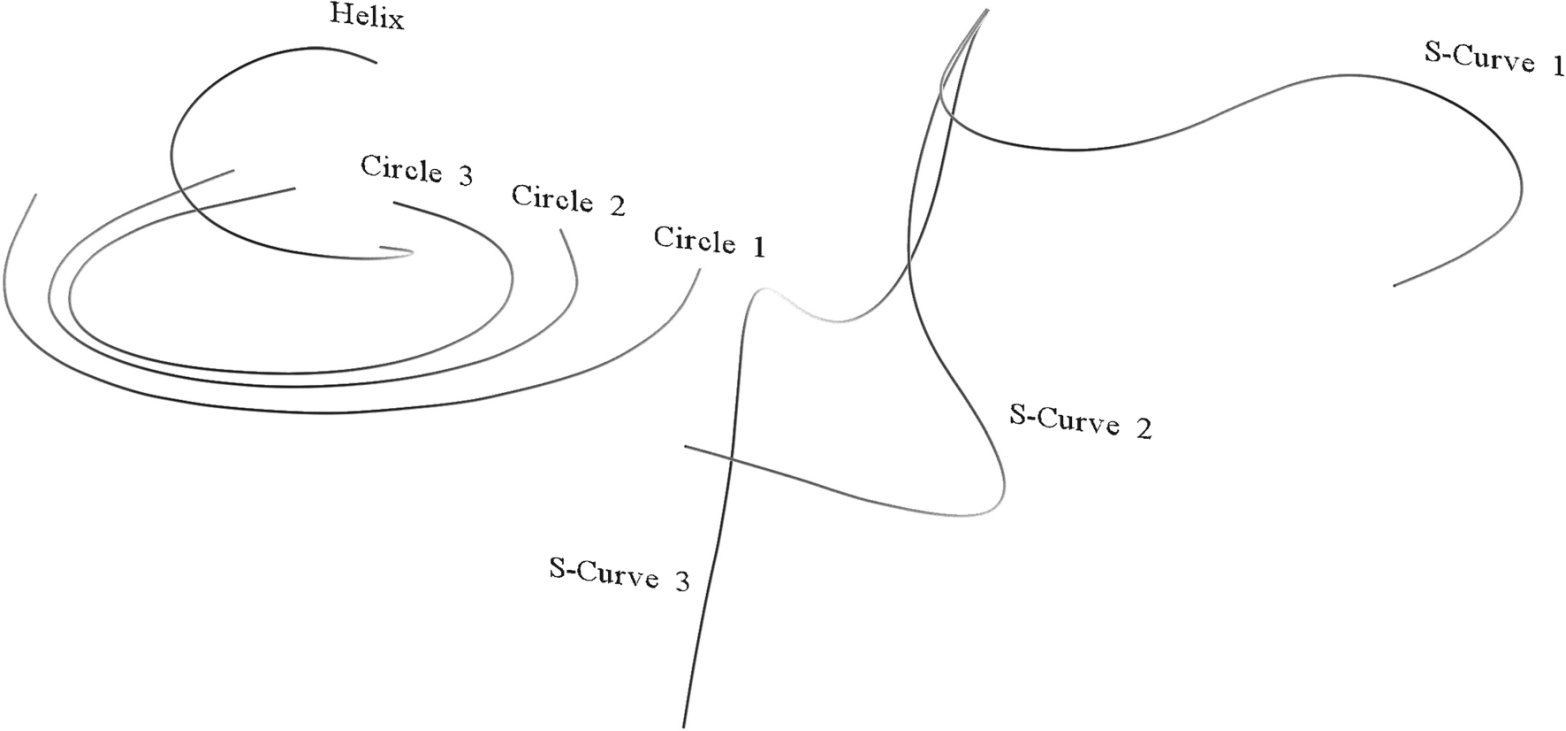
3D experiment with the fiber: the segmented shapes from the CT scan of the circle, s-curve and helix measurements are shown

### 3D shape reconstruction accuracy

For the 3D shape experiments, we integrated the results of all previous experiments in our model. We made several measurements bending our fiber to different 3D shapes. The segmented shapes from the CT scan of the measurements, as shown in Fig. 6, were used as ground truth. The accuracies, shown in Table 2, depend on the complexity of the forms: for the circular shapes, we obtain an average error of ${e_{\text {avg}} \approx 0.5~\mathrm{mm}}$ and a maximal error of ${e_{\text {max}} \approx 1~\mathrm{mm}}$, whereas for s-curved and helical shapes, we get higher errors, especially for *s-curve 3* and *helix*.

Comparing our results with Khan [7], we obtained higher errors. But Khan evaluated a catheter of only 114 mm length in different configurations with weakly bending, constant or linear curvature and nearly no torsion. Also they did not tested their catheter in any form with singularities like s-curves. Hence, the results of our 3D experiments with high deflections using the 380 mm multicore fiber are nevertheless accurate and promising.

**Fig. 7:**
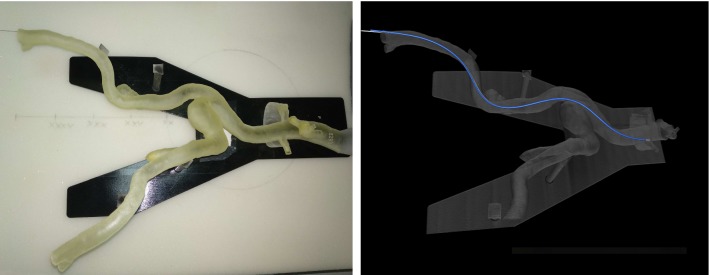
The first image shows the vessel phantom with the fiber inside, the second image the CT scan with the reconstructed shape (blue) and ground truth (white)

In the last experiment, we evaluated our model in a realistic endovascular scenario and inserted our fiber into a 3D printed vessel phantom, as shown in Fig. 7. Here, we obtained an average error $e_{\text {avg}}= 1.13~\mathrm{mm}$ and maximum error $e_{\text {max}}= 2.11~\mathrm{mm}$, which indicates an accurate reconstruction. This is also visible in the right image of Fig. 7: the reconstructed shape, represented by the blue line, fits almost perfectly to the ground truth of the CT scan. We also provided a video as electronic supplementary material, which shows further views.

## Conclusion

We presented an optimized shape sensing model with multicore fibers for flexible instruments. We conducted a detailed error analysis for every shape reconstruction step. The main error sources of shape sensing with multicore fibers are corrupted reference wavelengths for the wavelength shift computation, direction angles changed by the twist present in the fiber and curvature values, which are distorted by using an inaccurate photo-elastic coefficient or incorrect radii. This indicates that two calibration measurements need to be done for every fiber. The first one is used to determine the Bragg wavelength $\lambda _\mathrm{b}$ with $\kappa = 0$, the second one to get the twist angle $\varphi _{\text {twist}}$ where $\kappa \ne 0$ and the curvature scale factor. Further factors influencing the shape are the equations defined by the used core configuration, the curvature and angle interpolation and the reconstruction algorithm.

Furthermore, we evaluated the accuracy of our model with 3D measurements in a CT scanner. We received the accuracies $e_{\text {avg}} \approx 0.35$ to $1.15\,\mathrm{mm}$ and $e_{\text {max}} \approx 0.75$ to 7.53 mm. Finally, we tested our fiber system in an endovascular scenario and obtained high accuracies ($e_{\text {avg}}= 1.13 \,\mathrm{mm},\; e_{\text {max}}= 2.11 \,\mathrm{mm}$). These experiments show promising results for using multicore fibers for shape sensing of catheters.

In future work, we aim to enable a full endovascular catheter navigation. For this purpose, we plan to combine the reconstructed shape obtained by the multicore fiber with the position and orientation of an electromagnetic tracking system. Also, the experiments were conducted under ideal conditions and we want to analyze our model in more realistic scenarios and to compensate other effects that may occur, such as dynamic change in the twist angle during an endovascular procedure. For this problem, using helically wrapped fibers as fiber system might be a better choice.

## Electronic supplementary material

Below is the link to the electronic supplementary material.

Supplementary material 1 (mp4 2598 KB)
